# Enumeration Method for *Pseudomonas aeruginosa* in Water with High Bacterial Background Flora

**DOI:** 10.3390/microorganisms14091973

**Published:** 2026-09-07

**Authors:** Elfy Ly, Tess M. Hiemink, Sharona de Rijk, Karola Waar, Franciska M. Schets, Lucía Hernández Leal, Mark C. M. van Loosdrecht, Hetty Blaak, Heike Schmitt

**Affiliations:** 1 Wetsus, European Centre of Excellence for Sustainable Water Technology, 8911 MA Leeuwarden, The Netherlands; e.ly@tudelft.nl (E.L.); tesshiemink@hotmail.com (T.M.H.);; 2 Department Biotechnology, Delft University of Technology, 2629 HZ Delft, The Netherlands; 3 Centre for Zoonoses and Environmental Microbiology, Centre for Infectious Disease Control, National Institute for Public Health and the Environment (RIVM), 3720 BA Bilthoven, The Netherlandshetty.blaak@rivm.nl (H.B.); 4 Certe Medical Microbiology, 9728 NZ Groningen, The Netherlands; k.waar@certe.nl

**Keywords:** selective medium, chromogenic agar, wastewater, swimming ponds, performance characteristics, environmental monitoring, method validation

## Abstract

Reliable detection of *Pseudomonas aeruginosa* (PA) in water is essential for public health water quality assessments, yet culture-based methods remain challenged by high background flora. This study compared the performance of mPA-C agar and the commercial RAPID’ *P. aeruginosa* (RAPID PA) agar in (ozone-) treated wastewater effluent and (spiked) swimming pond water and evaluated how presumptive colony colour definitions and confirmation steps affected performance characteristics. mPA-C showed strong matrix dependence and high observer variability due to a broad colour colony variation (of both PA and non-PA). RAPID PA produced clearer and more consistent pigmentation with relatively limited background growth and performed more consistently across matrices. Expanding the presumptive colony colour definition significantly improved sensitivity of RAPID PA with minimal loss of specificity. A quick subculturing step on RAPID PA for visual confirmation further enhanced specificity and markedly reduced false-positive rates. Extended incubation to 48 h increased performance only for swimming ponds. RAPID PA performed more consistently across matrices. Overall, using RAPID PA with expanded colour criteria and subculturing as a low-effort confirmation step enabled effective culture-based detection of *P. aeruginosa* in high-background-flora water samples. This medium represents a practical and robust alternative for routine monitoring.

## 1. Introduction

Ensuring microbiological safety of water is essential for protecting public health, as water can serve as an important exposure route for opportunistic pathogens. One such pathogen of concern is *Pseudomonas aeruginosa*, a Gram-negative bacterium that is clinically significant yet also widely distributed in both natural and engineered environments. It is frequently detected in surface waters [1,2], artificial and natural swimming ponds [3,4], drinking water systems [5,6,7], and both raw and treated wastewater [8,9]. Tertiary treatment steps such as ozonation are increasingly applied to reduce microbial and chemical contaminants. However, *P. aeruginosa* has been reported to survive ozonation and even regrow during subsequent storage [10]. Beyond wastewater, the bacterium has also been detected on swimming pool equipment [11] and water-filled hospital materials and medical devices [12]. Its ecological versatility enables *P. aeruginosa* to persist in a wide range of aquatic habitats, as well as in soil, sediment, and biofilm-coated surfaces. *P. aeruginosa* is highly adaptable and capable of surviving under diverse environmental conditions including temperatures from 4 to 50 °C [13], varying nutrient availability [14,15], oxygen levels [16], and pH values from 5 to 9 [17].

*P. aeruginosa* is not part of the healthy human microbiota [18]; therefore, exposure through contaminated water can lead to a wide range of infections, including otitis externa, skin rashes, keratitis, urinary tract infections, pneumonia, and, in immunocompromised patients, life-threatening systemic infections [2,3,4,19,20]. Treatment of such infections is challenging due to the bacterium’s intrinsic and rapidly acquired resistance mechanisms [21,22,23,24]. The combination of environmental persistence, infection potential, and resistance to treatment highlights the importance of effective monitoring in water quality management.

Despite this relevance, current European bathing water regulations include *Escherichia coli* and intestinal enterococci as mandatory microbiological parameters, but not *P. aeruginosa* [25]. Some national frameworks, including those in the Netherlands, do include or investigate *P. aeruginosa* monitoring in specific water bodies such as swimming pools and artificial swimming ponds [26].

For the detection and enumeration of *P. aeruginosa* in aquatic environments, two reference methods currently exist. The first is a “colony count” method based on membrane filtration followed by culture on semi-selective CN agar [27], which is suitable for low-background waters such as bottled water and drinking water, but not for high-background sources such as swimming ponds. The second is a “most probable number” (MPN) method [28], which can handle high-background samples but does not yield colonies for further characterization. A former Dutch national standard [29,30] described a colony count method using mPA-D agar for high-background waters, but this standard has expired. A modified version, mPA-C agar, remains in use under the Australian/New Zealand standard [31]. However, no international reference method currently exists for enumerating *P. aeruginosa* in high-background waters using membrane filtration, such as swimming pond water [26]. Moreover, in practice, mPA-based media require considerable expertise and confirmatory testing to differentiate *P. aeruginosa* from morphologically similar species [26].

This study was driven by the practical need for a reliable enumeration method for *P. aeruginosa* in swimming ponds, where high background flora complicates detection. The aim was to establish a practical and accurate method suitable for such waters, while also being applicable to other water types with high microbial loads, such as treated wastewater. Beyond routine monitoring, the method may also support public health investigations, for example in cases of *P. aeruginosa*-related otitis externa outbreaks linked to recreational surface waters. The yield and categorical performance characteristics—specificity, sensitivity, false-positive rate, false-negative rate, and selectivity—of RAPID’*P. aeruginosa* agar (RAPID PA) were determined and compared with those of mPA-C agar, following the requirements of [32]. The evaluated water samples included naturally *P. aeruginosa*-contaminated waters such as secondary and ozone-treated wastewater, as well as (spiked) swimming pond water.

## 2. Material and Methods

### 2.1. Sampling Procedures and Sample Types

Sampling was performed between May 2023 and February 2024 in the Netherlands (Table 1). Grab samples were collected from various aquatic environments, including wastewater effluent of a wastewater treatment plant (WWTP) with secondary treatment, ozone-treated wastewater effluent, and swimming ponds. The effluents were derived from two WWTPs in the Netherlands, serving 106,285 and 48,685 inhabitants, respectively [33]. Ozone treatment of WWTP effluent was carried out in a lab-scale ozone reactor. The setup and procedure of this reactor are described in detail in a parallel study [34]. Because *P. aeruginosa* has been reported to survive ozonation and potentially regrow during subsequent storage [35], ozone-treated wastewater samples were also stored for seven days at room temperature under artificial light with continuous mixing to assess potential regrowth. Swimming pond samples were divided in two portions, one of which was spiked before analysis and the other part analysed “as is”. The reason for spiking was that *a priori P. aeruginosa* levels were not known. For spiking, either WWTP influent was used or a mix of six morphologically distinct *P. aeruginosa* isolates, four of which had been isolated from WWTP influent on mPA-C agar (BD, Franklin Lakes, NJ, USA) and two on RAPID’*P. aeruginosa* agar (Bio-Rad Laboratories, Marnes-la-Coquette, France). For analysis of performance characteristics, swimming pond samples had to be spiked (except for one of the samples) because of absent or low natural contamination. All samples were collected in sterile bottles and analysed within 24 h after sampling.

### 2.2. Agar Selection and Preparations

Non-selective Luria–Bertani (LB) agar was prepared in-house and Tryptone Soy Agar (TSA) was obtained pre-poured (Oxoid, Thermo Scientific, Landsmeer, The Netherlands). BD BBL™ mPA-C Agar (BD, Franklin Lakes, NJ, USA) was obtained as a dehydrated powder and prepared according to the manufacturer’s instructions. RAPID’*P. aeruginosa* (RAPID PA) agar (Bio-Rad Laboratories, Marnes-la-Coquette, France) was similarly obtained as a dehydrated powder and prepared according to the manufacturer’s instructions. Colonies exhibiting pigmentation characteristic of *P. aeruginosa* were classified as presumptive colonies.

For mPA-C agar, this is a brown-to-black or green-black colour based on the former Dutch standard and the Australian/New Zealand standard [30,31,36], or tan to dark-brown in colour with dark-brown centres or pink with nucleated centres or surrounded by an amber halo according to Brodsky and Ciebin (1978) [37]. In addition, de Vicente et al. (1986) described typical *P. aeruginosa* colonies as flat and dry, ranging in colour from greenish-grey with a dark centre to black with or without a greenish rim, or dark brown without a rim and more rounded in shape [38]. Based on these descriptions, brown, green, black and purple were selected as the presumptive target colours for presumptive colonies on mPA-C agar in this study.

For RAPID PA agar, presumptive *P. aeruginosa* colonies are defined by the manufacturer as blue to blue-green in colour [39].

### 2.3. Analysis of Colony Colours Associated with Target and Non-Target Species

As part of the evaluation of agar performance, colony colour and morphology were systematically recorded. To assess the relation between manufacturer-, standard- or literature-indicated colony characteristics and the bacterial species recovered, all colonies grown on mPA-C and RAPID PA agar were categorized by colour tones for easier interpretation (Table S1) [30,31,36,37,38,39]. Colonies displaying two colours were assigned to the darker predominant colour (e.g., blue-brown colonies were classified as brown). Based on this evaluation, on top of the previous descriptions, additional colour categories were defined that determined the colours of presumptive *P. aeruginosa*. The performance characteristics of these new colour categories were evaluated against the previously described colour categories.

### 2.4. Culture of Water Samples

In total, 20 water samples were filtered through filter membranes (0.45 µm pore size, 47 mm diameter, Millipore S.A.S., Molsheim, France) by vacuum filtration. A range of at least three different volumes was used depending on sample types (Table 1). Volumes were based on preliminary experiments (WWTP effluents) and *P. aeruginosa* concentrations in the spike material (swimming ponds) to ensure countable colony numbers. Filter membranes were placed on mPA-C and RAPID PA agar for incubation at 41.5 ± 1 °C for 21 ± 3 h.

### 2.5. Determination of Performance Characteristics

**Figure 1:**
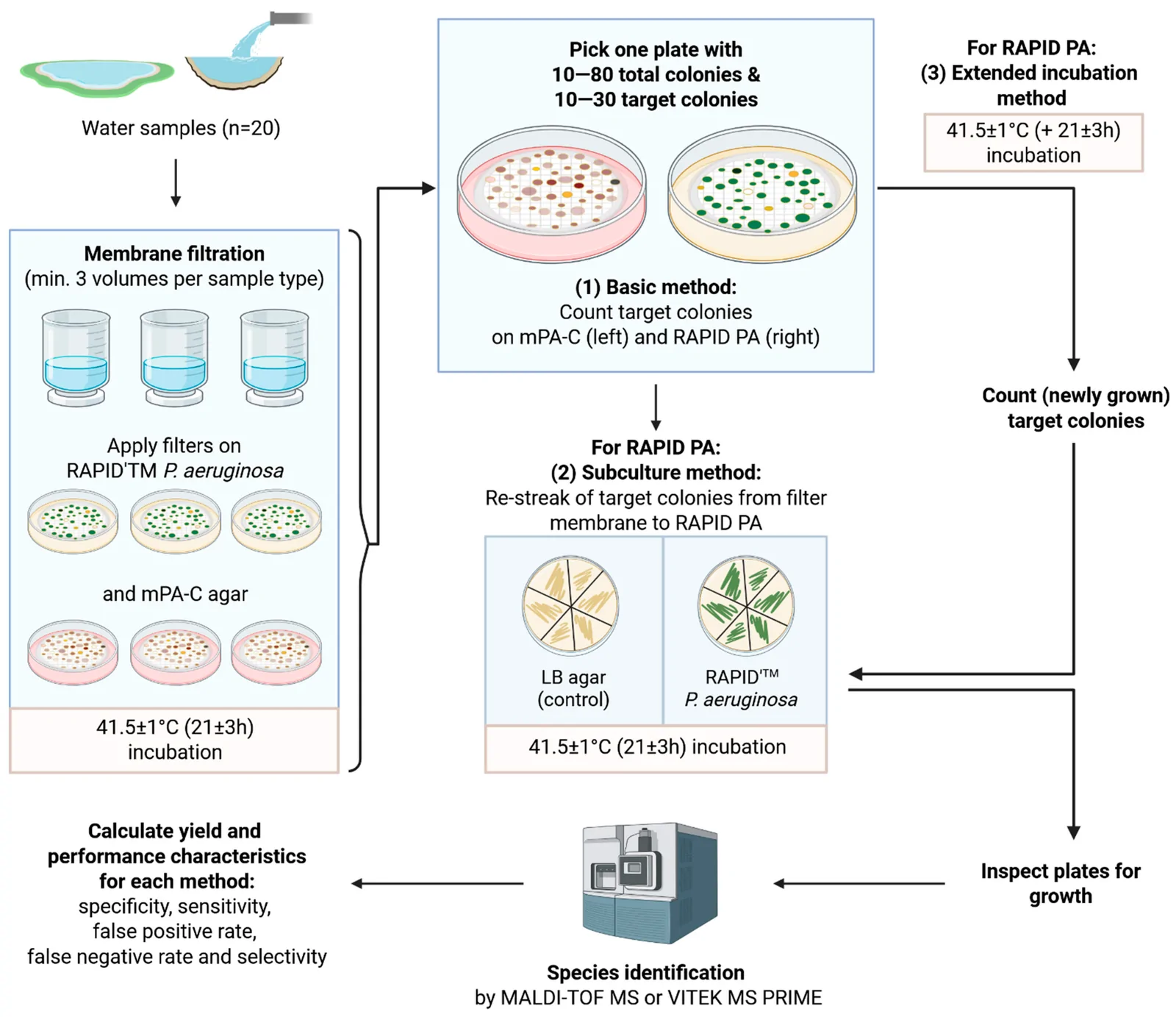
Schematic overview of the experimental procedures. Workflow for sample analysis, including key performance characteristics.

Categorical performance characteristics were determined as described in [32] (Figure 1). From each filtration series of one sample per medium, one filter with 10–80 total colonies and 10–30 presumptive colonies was selected. From this filter, all colonies—both presumptive and non-presumptive colonies—were subcultured on the corresponding agar for purification with a small streak (max. eight colonies per plate), followed by incubation at 41.5 ± 1 °C for 21 ± 3 h. Isolates were then subcultured on LB agar or TSA. Since some strains failed to grow on RAPID PA agar during subculturing, for nine (ozone-) treated wastewater samples colonies were also subcultured directly from the filters on non-selective LB agar, i.e., in parallel to subculture on RAPID PA agar and under the same incubation conditions (41.5 ± 1 °C for 21 ± 3 h), to allow identification of strains that were capable of growing on the filter but not capable of growing on RAPID PA agar upon subculture.

In total, 400 and 305 isolates were recovered from (ozone-) treated wastewater samples on mPA-C and RAPID PA agar, respectively. From (spiked) swimming pond samples, 462 and 407 isolates were recovered on mPA-C and RAPID PA agar, respectively. Matrix-assisted laser desorption/ionization time-of-flight mass spectrometry (MALDI-TOF MS) was used for isolate identification as it provides rapid and reliable confirmation of *P. aeruginosa*, minimizing ambiguity compared to conventional physiological or biochemical assays [40]. Colonies isolated from (ozone-) treated wastewater were analysed using VITEK^®^ MS PRIME (bioMérieux, Marcy-l’Étoile, France) by CERTE, IZORE (Medical Microbiology and Infection Control, Fryslan, Leeuwarden, The Netherlands) using database Version 3.2 for spectrum classification. Colonies isolated from swimming ponds were identified using a MALDI-TOF Bruker Microflex LT/SH and using BDAL 12.0.0.0 and SR 1.0.0.0 reference libraries (Bruker Daltonics, Bremen, Germany). Presumptive colonies that were confirmed as *P. aeruginosa* species with MALDI-TOF with either of the two methods are defined here as confirmed colonies.

**Figure 2:**
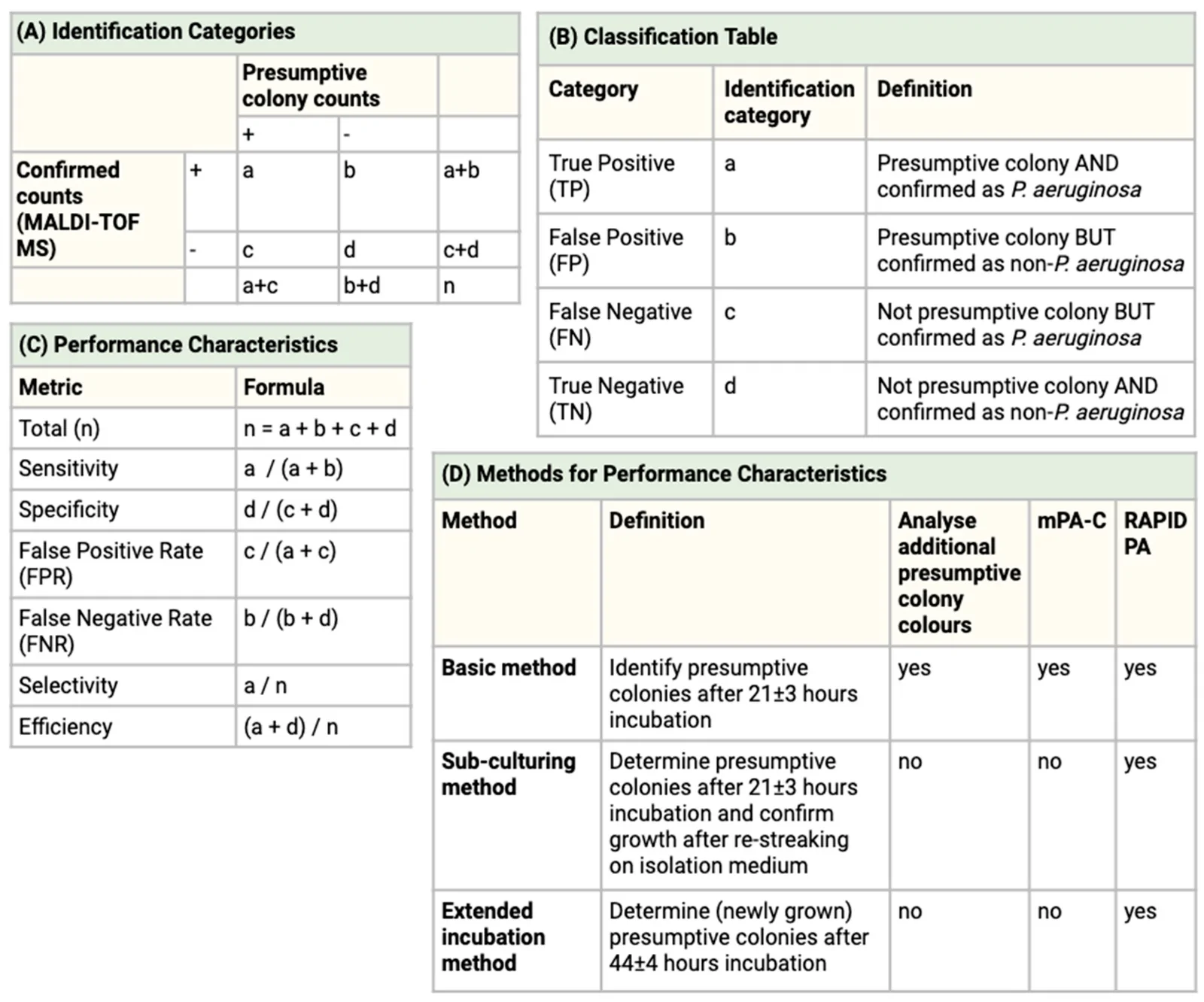
Calculations of performance characteristics according to and adapted from ISO:13843:2017 [32].

To determine performance characteristics of mPA-C and RAPID PA agar, each colony was classified as true positive, false positive, true negative, or false negative (Figure 2A) based on two or three different definitions of “presumptive” based on colony colour as described (Table S1) [30,31,36,37,38,39], and confirmation by MALDI-TOF MS (Figure 2B). For each different colour group chosen as the definition of presumptive, these classifications were used to calculate sensitivity, specificity, false-negative rate, false-positive rate, selectivity and efficiency (Figure 2C). For both agars and each definition of presumptive based on colony colour, performance characteristics were evaluated following the standard culture procedure, in which presumptive colonies are identified based on different colour categories after 21 ± 3 h of incubation (further defined as the basic method). For RAPID PA, performance characteristics were evaluated for two additional methods (Figure 2D).

Based on the best-performing colour category defining “presumptive” colonies, two alternative methods were assessed independently to determine whether performance could be improved beyond the basic method: a subculture method, in which presumptive colonies were re-streaked onto the isolation medium, and an extended incubation method, whereby plates were incubated for an additional 21 ± 3 h (total incubation time: 44 ± 4 h), as recommended by the manufacturer.

The subculture method was evaluated following the observation that some presumptive colonies failed to grow or lost their characteristic appearance upon re-streaking. This method was therefore expected to reduce false positives and increase specificity. In contrast, the extended incubation method allows additional colony development, enabling *P. aeruginosa* colonies with initially non-target morphologies to acquire characteristic features, potentially reducing false negatives and increasing true positives and sensitivity. Each method was applied independently and compared to the basic method.

Enhancements of subculturing or extended incubation were not applied on mPA-C for performance characteristics. Subculturing of presumptive colonies on mPA-C did not make a difference, and extending the total incubation period to 44 ± 4 h resulted in fungal overgrowth, which hindered colony assessment and complicated interpretation of presumptive colonies (Figure S1). Therefore, these enhancements were only applied to RAPID PA.

### 2.6. Data Analysis

Data analysis was done using R Studio version 4.2.1 [41] and plots were constructed using the ggplot2 package (v 3.5.1) [42]. For the heatmaps, colony colour and species identification, data were summarized by counting the frequency of each colour–species combination. Heatmaps were generated using the pheatmap package (v 1.0.13) in R to visualize the distribution of colony colours across species [43]. Significance of the effect of method changes as compared to either the reference method or a selected method on method sensitivity and specificity is tested through application of the McNemar’s test with continuity correction where appropriate using the stats package (v 4.6.0). The agreement of the colour rating by different people was analysed by letting four people assign colours to colonies on photographs of exemplary plates (two mPA-C and two RAPID-PA plates), for ten colonies per plate each, using the colour groups as specified in Table S1 [30,31,36,37,38,39]. The congruence of colour assignment was tested with Fleiss’ kappa test using the irr package (v 0.85).

## 3. Results

### 3.1. Colony Colour Profiles of P. aeruginosa and Background Flora on mPA-C and RAPID PA Agar

All colonies on the filter membrane recovered from (ozone-) treated wastewater and (spiked) swimming pond samples were assessed for colony colour and species, revealing matrix-dependent variations in pigmentation for both agars. Across all sample types, *P. aeruginosa* colonies displayed more consistent and distinctive pigmentation on RAPID PA agar than on mPA-C agar.

**Figure 3:**
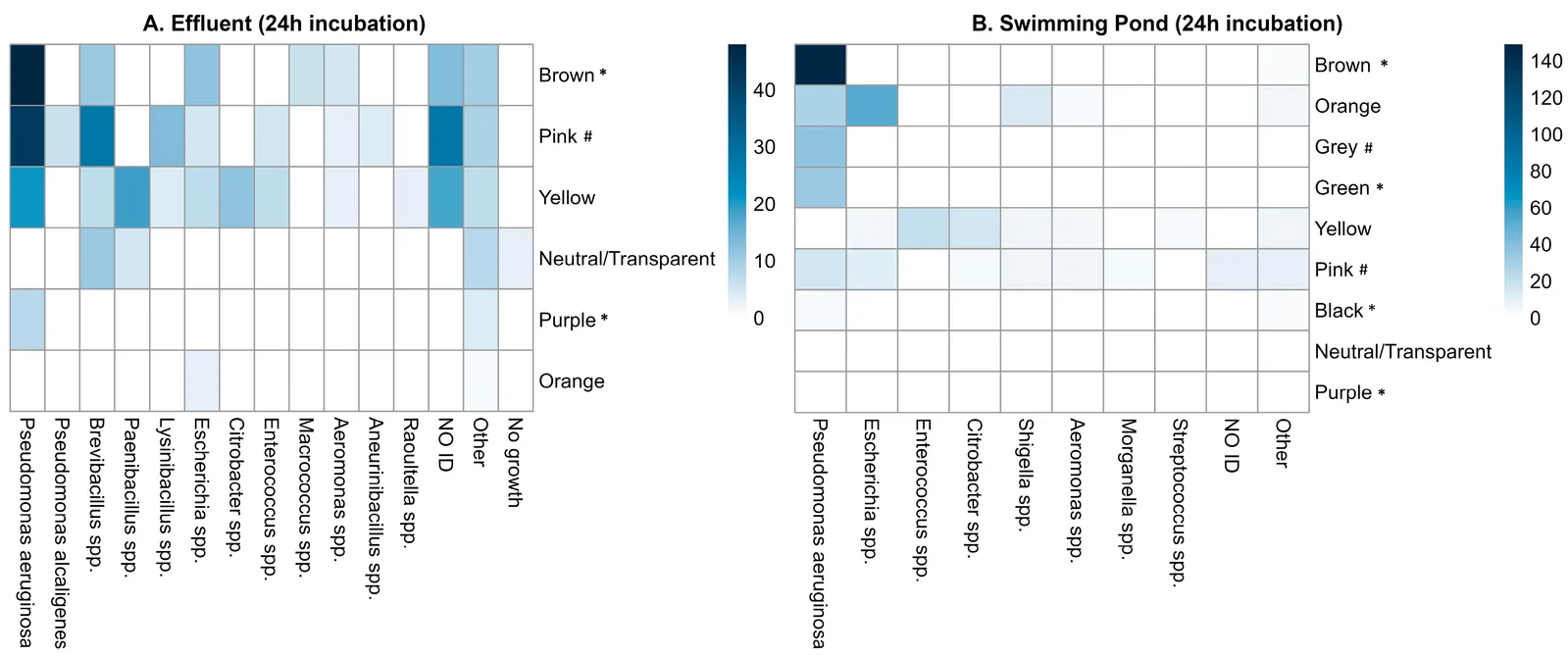
Heatmaps of colony colour tone distribution by bacterial genus on mPA-C agar after 21 ± 3 h incubation. Heatmaps show the distribution of colony colour tones with genus-level identifications where colour intensity indicates the number of colonies observed (from white to blue). Panels show genus-level identifications of colonies from: (**A**) (ozone-) treated wastewater effluent (*n* = 10) and (**B**) (spiked) swimming pond water (*n* = 10). Colours marked with asterisks indicate presumptive colonies according to the manufacturer. Colours indicated with hashtags (#) are included as presumptive colony colours for further analysis.

On mPA-C agar, *P. aeruginosa* colonies showed a broader and more variable colour spectrum across both matrices than the expected brown, black, green and purple tones described before. Although brown remained the predominant colour for both matrices (Figure 3A,B), additional hues were observed: in (ozone-) treated wastewater, *P. aeruginosa* colonies frequently appeared pink and often yellow (Figure 3A and Figure S1), while in (spiked) swimming pond samples, colonies additionally presented as orange, grey, and pink (Figure 3B and Figure S2). However, pink, yellow and orange pigments were also frequently produced by non-*P. aeruginosa* species, which could reduce the reliability when used as the presumptive colour colony on mPA-C agar.

**Figure 4:**
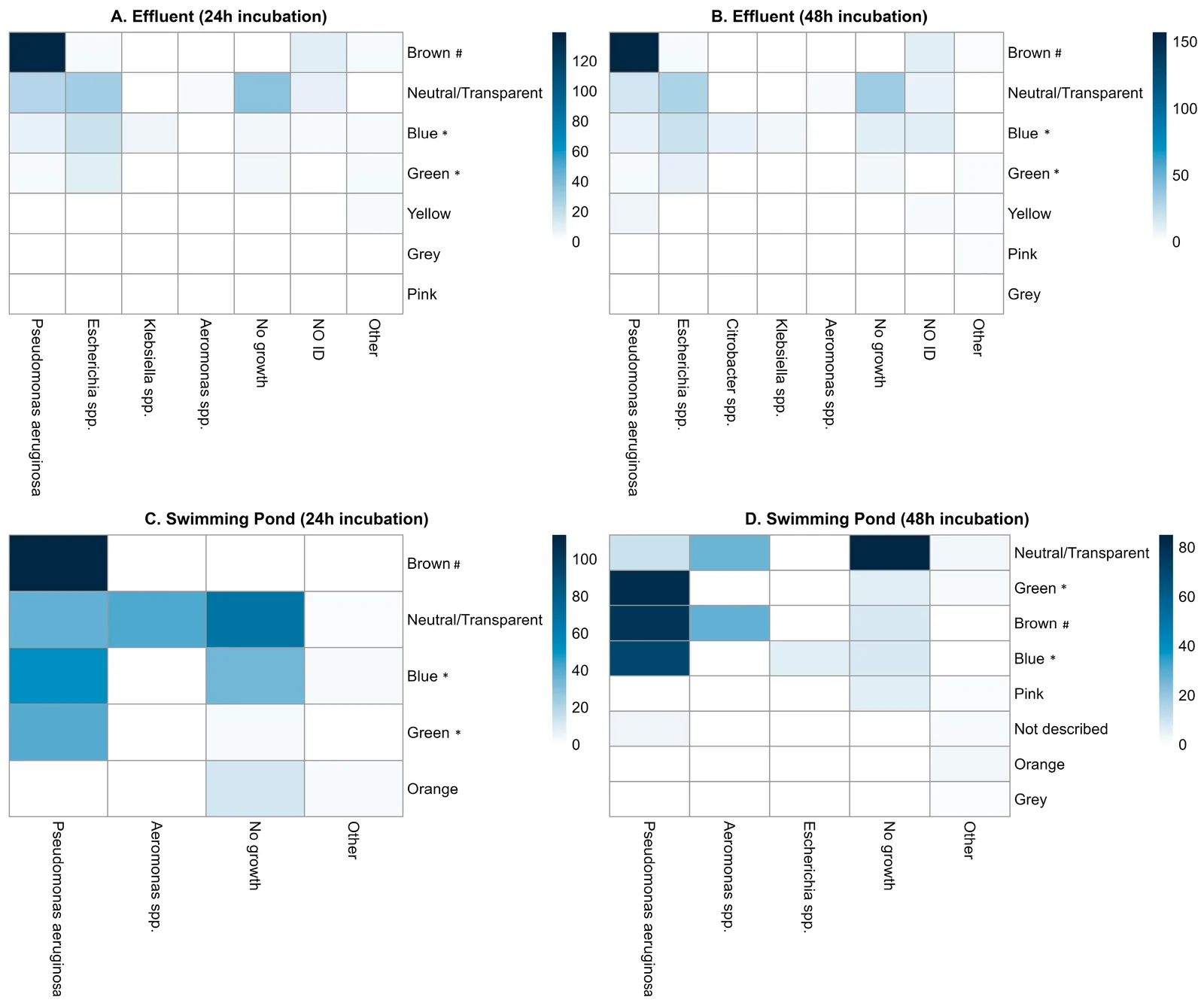
Heatmaps of colony colour group distribution by bacterial genus on RAPID PA agar after 24 h and 48 h incubation. Heatmaps show the distribution of colony colour tones with genus-level identifications where colour intensity indicates the number of colonies observed (from white to dark blue). Panels show genus-level identifications of colonies from (ozone-) treated wastewater effluent (**A**) after 21 ± 3 h incubation (*n* = 10) and (**B**) after 44 ± 4 h incubation (*n* = 10); and (spiked) swimming pond water (**C**) after 21 ± 3 h incubation (*n* = 10) and (**D**) after 44 ± 4 h incubation (*n* = 10). No growth signifies failure to grow after subculture on RAPID PA agar. “Not described” colours were impossible to describe due to overlap of colonies. Colours marked with asterisks indicate presumptive colonies according to the manufacturer. Colours indicated with hashtags (#) are included as presumptive colony colours for further analysis.

In contrast, on RAPID PA agar for (ozone-) treated wastewater, *P. aeruginosa* colonies were predominantly blue/green with brown (categorized as brown) with minimal overlap from other genera after 21 ± 3 h incubation (Figure 4A). After 44 ± 4 h incubation, these colonies became bigger and darker, and neutral/transparent colonies mainly turned green, blue or brown (Figure 4B and Figure S3). In (spiked) swimming pond samples, colour appearance varied depending on the spiking material: wastewater influent spiking produced a broader range of colony colours (Figure S4A), whereas a mixture of six *P. aeruginosa* strains showed more uniform pigmentation (Figure S4B). Overall, *P. aeruginosa* appeared as brown, blue, green and neutral/transparent colonies after 21 ± 3 h incubation (Figure 4C). Also here, after 44 ± 4 h incubation, *P. aeruginosa* colonies became bigger and darker, and neutral/transparent *P. aeruginosa* colonies mainly turned green, blue or brown (Figure 4D). Non-target species remained neutral or transparent and did not grow upon subculturing on RAPID PA agar. Notably, across both matrices, *P. aeruginosa* produced brown-toned colonies, a colour not indicated by the manufacturer, who describes *the species* as blue to blue-green [39].

### 3.2. Bacterial Composition of Various Presumptive Colonies on mPA-C and RAPID PA Agars After 24 h Incubation

Based on the heatmaps, additional colour categories were defined on top of the previous described colours for presumptive colonies (Table S1) [30,31,36,37,38,39]. For mPA-C agar, the conventionally described presumptive colours—brown, green, black, or purple (category A)—were expanded by adding grey colonies (category B) and further extended to include both grey and pink colonies (category C). Yellow was not added as a presumptive colony colour because of extensive non-*P. aeruginosa* growth and, in swimming pond samples, the absence of yellow coloured *P. aeruginosa colonies*. For RAPID PA agar, in addition to the manufacturer-specified blue-green colonies (category A), brown colonies were included as an expanded presumptive category (category C). The bacterial composition of colonies falling into these new presumptive colour categories were evaluated against the previously described colour categories. The proportion and absolute number of confirmed *P. aeruginosa* colonies varied markedly between media, matrices, and the colour categories defined for presumptive colonies.

**Figure 5:**
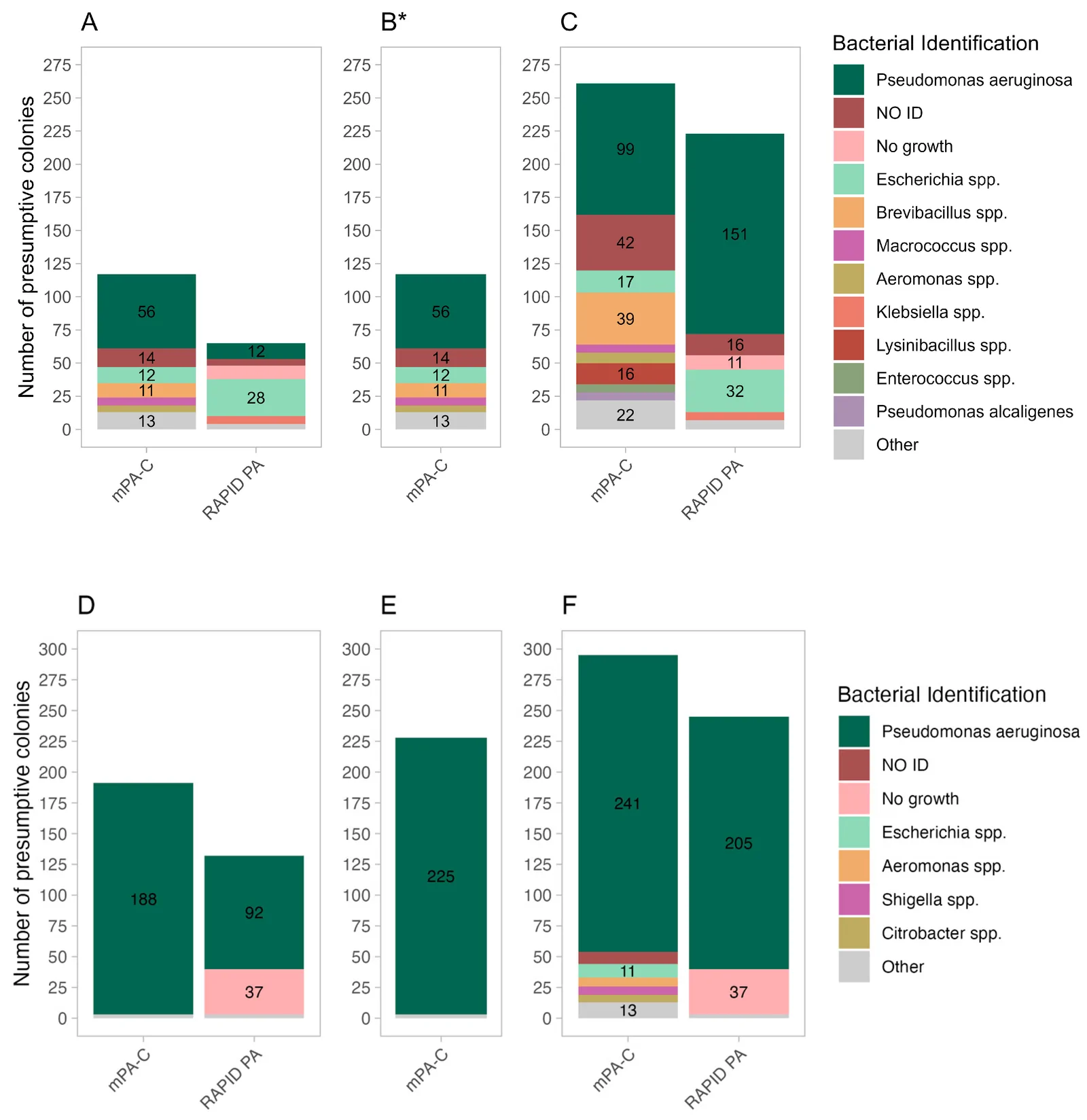
Genus-level classification of presumptive *P. aeruginosa* isolates from (ozone-) treated wastewater (**A**–**C**) and (spiked) swimming pond samples (**D**–**F**) on mPA-C and RAPID PA agars after 21 ± 3 h incubation, identified by MALDI-TOF MS. Panels (**A**,**D**) show the results for manufacturer- or literature-based presumptive colony colours—brown, green, black and purple for mPA-C agar, and blue and green for RAPID PA agar; panels (**B**,**E**) include grey as presumptive colony colour on mPA-C agar; and panels (**C**,**F**) include grey and pink as presumptive colony colours on mPA-C agar and brown as presumptive colony colour on RAPID PA agar. Bars indicate total isolates per agar, with genus-level classifications shown by colour. The number of isolates per genus (≥10) is shown within each bar segment. “No growth” denotes colonies that failed to regrow upon subculturing on the isolation medium; genera with <5 isolates were grouped as “Other.” * No grey colonies were observed; therefore, panels (**A**,**B**) are identical.

On mPA-C agar, for ozone-treated wastewater (Figure 5A–C), adding grey as a presumptive colony colour caused no change in the number or confirmation rate of *P. aeruginosa*—as no grey colonies were present—whereas including pink as a presumptive colony colour nearly doubled the number of confirmed isolates from 56 to 99 across all samples. However, the proportion of *P. aeruginosa* decreased (47.9% to 37.9% of presumptive colonies) due to an increased number of non-presumptive colonies among the presumptive colonies. In swimming pond samples (Figure 5D–F), including grey as a presumptive colony colour slightly increased the numbers of confirmed *P. aeruginosa* counts from 188 to 225 (with conformation rates of 98.4% to 98.7% of presumptive colonies, respectively), while further inclusion of pink as a presumptive colony colour raised *P. aeruginosa* counts from 225 to 241 but reduced confirmation percentages from 98% to 82% of presumptive colonies, respectively, indicating greater interference from background flora.

On RAPID PA agar, for ozone-treated wastewater (Figure 5A,C), inclusion of brown colonies as presumptive colonies increased the number of confirmed *P. aeruginosa* from 12 to 151 (with confirmation rates 18.5% to 67.7% of presumptive colonies, respectively), markedly enhancing both numbers and proportion of confirmed *P. aeruginosa*. In swimming pond samples (Figure 5D,F), inclusion of brown colonies similarly improved the number of confirmed *P. aeruginosa* from 92 to 205 (with confirmation rates of 69.7% to 83.7% of presumptive colonies, respectively).

Overall, adding brown as a presumptive colony colour on RAPID PA agar enhanced both recovery and proportion of *P. aeruginosa* among presumptive colonies, whereas expanding the existing colour definition for presumptive colonies with grey or pink on mPA-C agar increased recovery without improving the proportion of *P. aeruginosa*.

As a range of different colours was included in the definition of presumptive colonies, the congruence of colour assignments was tested between four different persons. For mPA-C agar, Fleiss’ kappa amounted to 0.11, while for colonies from RAPID PA agar, Fleiss’ kappa equaled 0.44. Disagreement between different individuals for RAPID-PA overall led to categorization differences for 2/20 colonies (i.e., the colour assigned by different individuals resulted in either “target” or “non-target” categorisation), while this was the case for 16/20 colonies on mPA-C agar.

### 3.3. Bacterial Composition of the Expanded Presumptive Colonies on RAPID PA Agar After Subculturing and Extended Incubation

**Figure 6:**
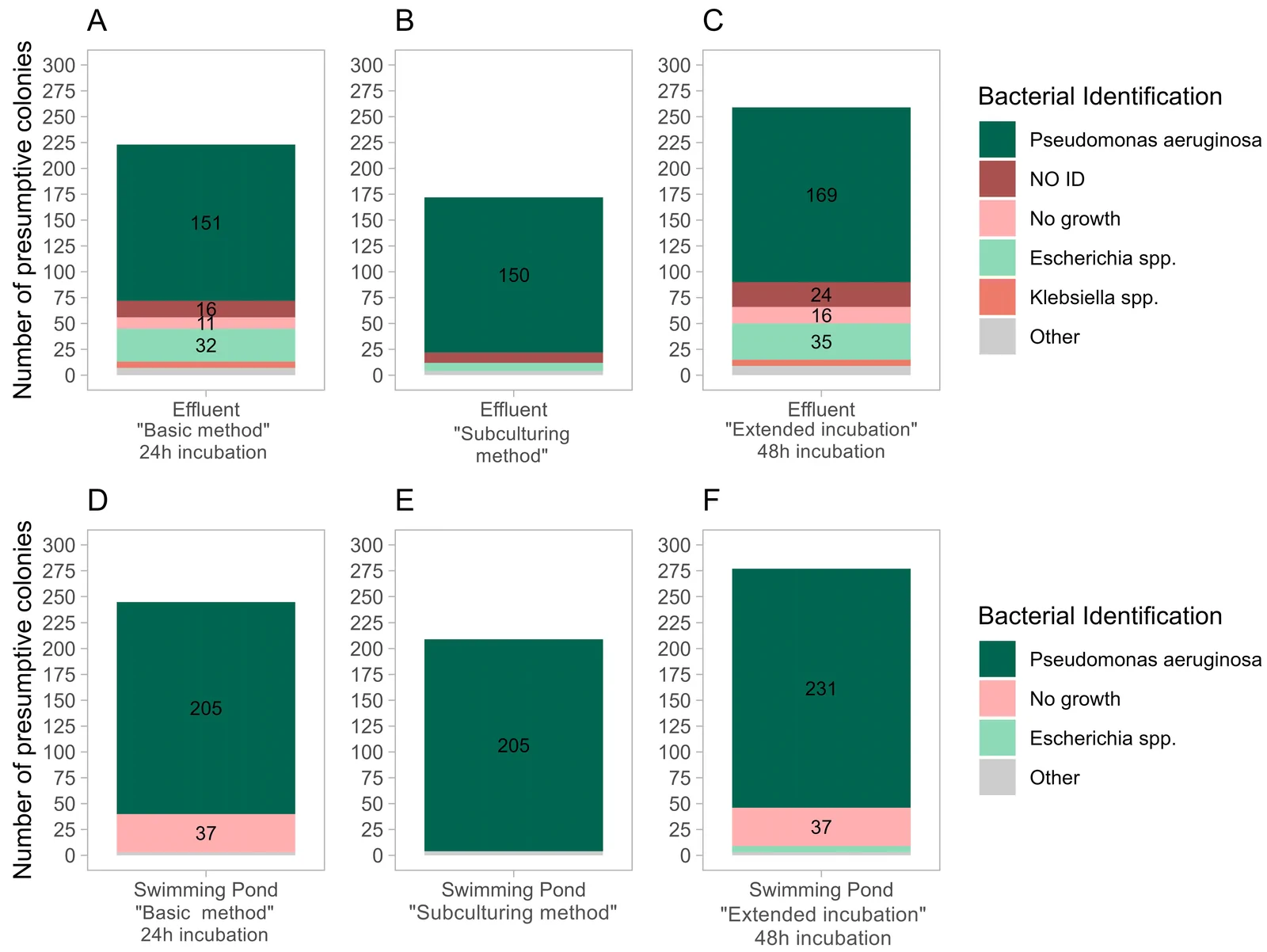
Genus-level classification of presumptive *P. aeruginosa* isolates (with colony colours blue, green and brown) from (ozone-) treated effluent and (spiked) swimming pond samples on RAPID PA agar after 24 h incubation, subculturing, and extended incubation. Panels (**A**–**C**) show (ozone-) treated effluent isolates, and panels (**D**–**F**) show (spiked) swimming pond isolates. Panels (**A**,**D**) show the results for presumptive colonies after 24 h incubation based on the expanded colony colour definition for presumptive colonies; these results correspond to those shown for RAPID PA agar in Figure 5C,F, respectively. Panels (**B**,**E**) show the results after subculturing presumptive colonies for growth confirmation, and panels (**C**,**F**) show the results based on the colony colour after extended incubation. Bars indicate total presumptive colonies per condition, with genus-level classifications shown by colour. Only genera with ≥5 isolates are shown individually; genera with fewer than five are grouped as “Other.” The number of isolates per genus (≥10) is indicated within each bar segment.

Using the expanded colour definition with blue, green and brown as presumptive colonies on RAPID PA agar (Figure 5C,F), two additional analyses were conducted: subculturing presumptive colonies on the isolation medium and an extended incubation up to 48 h. Both subculturing of presumptive colonies and extended incubation further improved the detection of *P. aeruginosa* on RAPID PA agar (Figure 6). In both matrices, subculturing reduced the total number of presumptive colonies because only colonies capable of regrowth were retained (Figure 6B,E). Extended incubation also increased the number of total presumptive colonies for both matrices but showed discrepant results with respect to the proportions of *P. aeruginosa* amongst the presumptive colonies (Figure 6C,F).

In (ozone-) treated effluent samples (Figure 6A–C), the total number of presumptive colonies reduced from 223 to 172 after subculturing and increased the confirmed *P. aeruginosa* proportion from 66.7% to 87.2%. The total number of *P. aeruginosa* identified remained nearly identical (150 vs. 151). Extended incubation instead of subculturing increased the total number of presumptive colonies from 223 to 264 and confirmed *P. aeruginosa* colonies from 151 to 169 leading to a decreased confirmation rate from 66.7% to 64.0%.

In (spiked) swimming pond samples (Figure 6D–F), the total number of presumptive colonies was reduced from 245 to 209 upon subculture, while the confirmation rate was increased from 83.7% to 98.1%. Extended incubation increased the confirmed *P. aeruginosa* colonies from 205 to 231 and increased the confirmation rate from 83.7% to 96.3%.

### 3.4. Performance Characteristics of mPA-C and RAPID PA Agar

Performance characteristics were determined for different colour categories defining presumptive colonies on mPA-C and RAPID PA agars as described above (Table 2): the standard definitions based on the literature and manufacturer descriptions (category A), inclusion of grey as presumptive colour for mPA-C agar (category B), and inclusion of grey and pink for mPA-C agar and brown for RAPID PA agar (category C). Performance characteristics were also assessed for the additional methods (subculturing and extended method) on RAPID PA agar as described, using blue, green and brown as presumptive colonies (Table 3).

On mPA-C agar (Table 2), considering grey colonies as presumptive (category B) did not change performance in (ozone-) treated effluent samples, as no *P. aeruginosa* of this colour were detected. Adding pink colonies as presumptive (category C) increased sensitivity but reduced specificity and efficiency due to a higher number of false positives. In (spiked) swimming pond samples, performance was already higher compared to (ozone-) treated effluent using the standard colour definition and including grey colonies as presumptive further improved sensitivity and reduced false negatives. Inclusion of pink colonies further increased sensitivity, but at the cost of lower specificity and more false positives. Overall, expanding presumptive colour definitions on mPA-C agar increased sensitivity and selectivity but particularly when pink colonies were included; specificity, efficiency and false-positive rates were compromised.

## 4. Discussion

This study evaluated the performance of mPA-C and RAPID PA agar for the detection of *P. aeruginosa* in water with high background flora, and examined how presumptive colony colour definitions, subculturing, and extended incubation affect performance characteristics. Overall, RAPID PA agar provided more consistent performance than mPA-C agar, particularly when brown colonies were included as presumptive targets and when a simple subculturing step was used to confirm growth. These findings highlight the interaction between medium selection, colour interpretation, and workflow steps in culture-based detection of *P. aeruginosa*.

### 4.1. Presumptive Colony Colour Definitions Require Refinement

Both media rely on colony colour for presumptive identification, but interpretation is not straightforward. For mPA-C agar, the presumptive colony definition reported in the literature covers a broad and partially ambiguous spectrum—from black and green to brown and purple [30,31,36,38]—making the interpretation highly observer-dependent. Our findings show that colour appearance is matrix-dependent: (spiked) swimming pond samples, with lower background flora and more uniform *P. aeruginosa* colonies due to spiking, produced a narrower colour range, whereas in treated wastewater, with higher microbial diversity, more variability in *P. aeruginosa* pigmentation was found. In contrast, *P. aeruginosa* species on RAPID PA agar produced more stable and distinct pyocyanin-associated blue-green and brown pigmentation, with markedly less background growth. This clearer colour differentiation reduced ambiguity and improved presumptive identification. Importantly, the assessments in this study were based solely on colours; incorporating colony morphology for the identification of *P. aeruginosa* as described in different studies, would introduce additional complexity, particularly for mPA-C agar where colony appearance is far more heterogeneous.

### 4.2. Presumptive Colony Colour Definitions Strongly Influence Agar Performance After 24 h Incubation

The choice of presumptive colours had a substantial impact on performance characteristics. For mPA-C agar, using only the colours described in previous studies (black, green, brown and purple) was inferior to expanded colour categories after 24 h incubation. Expanding the presumptive definition to include grey colonies (category B) and also to grey and pink colonies (category C) improved performances. Including both grey and pink colonies (category C) consistently resulted in significantly higher sensitivity, higher selectivity and lower false negatives but significantly reduced specificity and increased false-positive rates. In the context of screening water samples with high background flora, sensitivity and low false-negative rates are important, as false-negative findings directly lead to underestimation of *P. aeruginosa* presence, whereas false-positive presumptive colonies can be resolved through subsequent confirmation steps. The broader colour inclusion in category C therefore increased the likelihood of capturing true *P. aeruginosa* colonies at the cost of reduced specificity, a trade-off considered acceptable for initial detection. For RAPID PA agar, expanding the presumptive definition to include brown colonies (category C) consistently improved performance relative to using only blue/green colonies as specified by the manufacturer (category A). Incorporating brown colonies substantially and significantly increased sensitivity and recovered many additional true *P. aeruginosa* isolates without substantially increasing false positives. With respect to ease of interpretation of the colour categories, the congruence of assigning colours to mPA-C agar between different individuals was low (Fleiss’ kappa < 0.2), while there was moderate agreement between different individuals when reading colours of colonies on filters on RAPID-PA agar (Fleiss’ kappa > 0.4), and these differences seldom led to differences in categorisation into target or non-target colonies—in contrast to mPA-C agar. Thus, ease of interpretation is much higher on RAPID-PA than on mPA-C agar.

### 4.3. Subculturing Improves Performance; Extended Incubation Is Less Effective for Effluent

Subculturing presumptive RAPID PA agar colonies on the isolation medium yielded the best performance across both matrices compared with basing presumptive colonies on colony colour alone. Most notably, it significantly increased specificity and markedly reduced the false-positive rate. This is because, apparently, non-*P. aeruginosa* species exist that form (presumptive) colonies on the membrane but are unable to grow on RAPID PA agar when re-streaked directly onto it. This may result from limited diffusion of selective agents in the agar through the membrane or due to nutrient carryover associated with other bacteria on the membrane that temporarily support growth.

Extended incubation showed matrix-dependent effects. In treated effluent, extending incubation resulted in only minor improvements over the basic 24 h method with expanded colour definitions and did not outperform subculturing, likely due to the higher background flora in this matrix. In contrast, in swimming pond water, extended incubation yielded better overall performance than subculturing, with higher sensitivity and efficiency and a lower false-negative rate. These gains were achieved at the cost of a slightly increased false-positive rate (remaining low at 3%) and a modest reduction in specificity.

Thus, for matrices with lower background interference, extended incubation represents an effective alternative to subculturing, whereas subculturing remains more suitable for complex matrices such as treated effluent. The stronger positive effect of subculture for effluents compared to swimming ponds, may result from higher levels, and different compositions of background flora. The abundant presence of non-*P. aeruginosa* species in effluents that can grow on membranes with presumptive colony colours as false positives but that are incapable of growing on RAPID PA directly, enables the use of this relatively simple subculturing method as confirmation.

### 4.4. RAPID PA Outperforms mPA-C Agar Across Matrices

When the best-performing methods for each medium were compared, RAPID PA consistently outperformed mPA-C agar. Without the optimizing method adaptations for RAPID PA agar but only considering the expanded colour definitions and interpreting filters at 24 h after incubation, RAPID PA agar already performed better than mPA-C agar in treated effluent. For swimming pond samples, both agars performed similarly using the basic method. Using the expanded colour definition, both subculturing and extended incubation with RAPID PA agar generated yet higher sensitivity, specificity, and efficiency than mPA-C agar with the best-performing colour definition (category C).

It should be noted that fungal overgrowth of mPA-C agar did not allow for a comparison of both agars at 48 h of incubation. Possibly, amendment of mPA-C agar with fungicides could assist in improving performance characteristics of mPA-C through extended incubation duration; however, this was not tested here.

The difference between methods was thus matrix-dependent. This matrix difference may be related to lower background flora in swimming ponds as well as by the use of spiked samples, as most swimming pond samples did not contain *P. aeruginosa*. Although the six isolates used for spiking were selected based on different morphology (four morphologies on mPA-C agar and two on RAPID PA agar), this limited the overall range of different *P. aeruginosa* morphologies considerably and led to more uniform morphologies, while (as our study confirms) *P. aeruginosa* can exhibit a wide morphological variation. Because of this, it was easier to correctly distinguish between *P. aeruginosa* and non-*P*. *aeruginosa* colonies in spiked swimming pond samples while colour-based identification was more challenging in effluents. Adding colour categories such as pink reduced false negatives, but dramatically increased false positives, illustrating the difficulty of balancing sensitivity and specificity with mPA-C agar. As the comparison of both methods in effluent is arguably more representative for a naturally diverse *P. aeruginosa* population, the better performance of RAPID PA agar in effluent supports the overall conclusion that RAPID PA agar represents a practical and efficient approach for detection of diverse *P. aeruginosa* phenotypes.

### 4.5. Practical Implications for Monitoring P. aeruginosa in Water

When interpretation based on presumptive colour is considered, including additional colour categories is essential for performance on both agars. The use of RAPID PA agar is favoured above the use of mPA-C agar, as mPA-C agar produced complex and heterogeneous colour patterns that were difficult to interpret reliably. For comparison of quantities of *P. aeruginosa* in multiple samples where minimizing false positives and high sensitivity is required, RAPID PA agar, combined with a simple subculturing step, outperformed mPA-C agar in both matrices. For quantification of microbiological water quality in public health settings, such as for swimming ponds, where minimizing false negatives and high specificity is essential, prolonged incubation on RAPID PA agar also provides a better alternative to subculturing. When using this method, it is important to score and compare filters at both 21 ± 3 and 44 ± 4 h for colony development. *P. aeruginosa* colonies tend to become very large and therewith overgrow and/or merge with other colonies, complicating interpretation. Photographing the membranes at 24 h can support accurate comparison of colony development. A practical limitation is that RAPID PA agar is a commercial medium and may not be universally available or aligned with international standardization, whereas mPA-C agar has long-standing use in ISO workflows.

## 5. Conclusions

RAPID PA agar combined with subculturing as a simple and low-effort confirmation step, provides a practical and effective approach for detecting *P. aeruginosa* in water matrices with high background flora, such as wastewater effluent. In water matrices with less background flora, prolonged incubations can be used instead of subculturing for confirmation. RAPID PA agar offers clearer pigmentation, greater selectivity, lower false-positive rates, and reduced observer-dependent interpretation as compared to mPA-C agar. Defining blue, green, and brown colonies as presumptive targets for RAPID PA agar and confirming growth by re-streaking markedly improved specificity and reduced false positives. While mPA-C agar performed similarly to RAPID PA agar in spiked swimming pond water without a confirmation step, its performance was matrix-dependent and less suitable for effluent samples. Overall, RAPID PA agar represents a robust alternative for routine monitoring of *P. aeruginosa*.

## Figures and Tables

**Table 1:** Overview of water sample types and conditions.

Scheme	*n*	Sampling Point	Storage	Spiked	Filtration Volume (mL)
**WWTP effluent**	6	WWTP with secondary treatment location A (4x) and B (2x), The Netherlands	-	-	3–10–30
**Ozone-treated wastewater effluent**	2	Lab-scale ozone set-up	-	-	3–10–100
**Ozone-treated wastewater effluent**	2	Lab-scale ozone set-up	7 days	-	20–30–40–50
**Swimming pond water**	9	Swimming pond A (2x), B (2x), C (1x), D (2x), E (2x), The Netherlands	-	Yes *	3–10–30–100
**Swimming pond water**	1	Swimming pond C, The Netherlands	-	-	3–10–30–100

* with diluted WWTP influent (sample A.1, B.1, C) or a mix of six *P. aeruginosa* strains in a final concentration of 200 CFU/L (sample A.2, B.2, D.1, D.2, E.1, E.2).

**Table 2:** Performance characteristics of mPA-C agar using different colony colour groups as presumptive colonies for *P. aeruginosa* detection after 24 h incubation in (ozone-) treated effluent and (spiked) swimming pond water. Presumptive colonies were classified as (A) literature-described colony colours (black, green, brown, purple), (B) including grey, and (C) including grey and pink. Significant changes in sensitivity and specificity in comparison to the reference category (A) are shown by *** (*p* < 0.001).

	(Ozone-) Treated Effluent			(Spiked) Swimming Pond Water		
	A	B *	C	A	B	C
Sensitivity (%)	46.7	46.7	82.5 ***	69.6	83.3 ***	89.3 ***
Specificity (%)	78.3	78.3	44.7 ***	98.4	98.4	71.9 ***
False-Negative Rate (%)	27.4	27.4	17.8	30.3	19.2	17.4
False-Positive Rate (%)	45.6	45.6	54.8	1.6	1.3	18.3
Selectivity (%)	16.6	16.6	29.4	40.7	48.7	52.2
Efficiency (%)	67.1	67.1	58.2	81.6	89.6	82.0
Samples	10			10		
Total Isolates	400			462		

* No grey colonies were observed; therefore, categories A and B for (ozone-) treated effluent yield identical values. On RAPID PA agar (Table 3), including brown colonies as presumptive colony colour (category C) markedly improved performance for both matrices compared with the standard colour definition (category A), with substantial increases in overall sensitivity, selectivity and efficiency, and reductions in false negative and positive rates. Incorporating a subculturing step of presumptive colonies further enhanced sensitivity and efficiency, particularly by reductions in false-positive rates for both matrices. In (ozone-) treated effluent, extended incubation improved its performance compared to the basic method (with expanded colour definition) but did not outperform the subculturing method. In (spiked) swimming pond water, extended incubation improved all characteristics. Compared to the subculture method, extended incubation resulted in higher sensitivity but higher false-positive rates and slightly reduced specificity.

**Table 3:** Performance characteristics of RAPID PA agar for *P. aeruginosa* detection in (ozone-) treated effluent and (spiked) swimming pond water. Presumptive colonies were classified as (A) manufacturer-defined colours (blue and green) and (C) including brown as presumptive colonies. Results are shown after 21 ± 3 h incubation, following subculturing for growth confirmation, and after 44 ± 4 h incubation. Significant changes in sensitivity and specificity are shown as: ^ns^ (*p* > 0.05), * (*p* < 0.05 and *p* > 0.01) or *** (*p* < 0.001). Changes in sensitivity and specificity in comparison to category A with 21 ± 3 h incubation are preceded by ^†^, in comparison to category C with 21 ± 3 h incubation are preceded by ^‡^, and in comparison to category C with subculturing are preceded by ^/§^.

**(Ozone-) Treated Effluent**				
	**A: Blue/Green**	**C: Blue/Green/Brown**		
	**21 ± 3 h Incubation**	**21 ± 3 h Incubation**	**Subculturing for Growth Confirmation**	**44 ± 4 h Incubation**
Sensitivity (%)	6.8	85.3 ^†^***	83.1 ^‡^*	81.9 ^‡ns/§ns^
Specificity (%)	62.5	56.2 ^†^*	93.0 ^‡^***	87.9 ^‡^***^/§ns^
False-Negative Rate (%)	67.4	26.5	20.1	22.0
False-Positive Rate (%)	80.0	27.1	5.8	9.7
Selectivity (%)	3.9	49.5	48.2	47.3
Efficiency (%)	30.2	73.1	87.2	84.4
Samples	10	10		
Total Isolates	305	305		334
**(Spiked) Swimming Pond Water**				
	**A: Blue/Green**	**C: Blue/Green/Brown**		
Sensitivity (%)	37.7	84.0 ^†^***	84.0 ^‡ns^	94.3 ^‡^***^/§^***
Specificity (%)	75.5	75.5 ^†ns^	97.6 ^‡^***	96.2 ^‡^***^/§ns^
False-Negative Rate (%)	55.3	24.1	19.7	7.3
False-Positive Rate (%)	30.3	16.3	1.9	3.0
Selectivity (%)	22.6	50.4	50.3	53.5
Efficiency (%)	52.8	80.6	89.4	95.1
Samples	10	10		
Total Isolates	407	407		430

## Data Availability

Supporting data can be found in the Supplementary Material. Further inquiries can be directed to the corresponding author.

## Supplementary Materials

The following supporting information can be downloaded at: https://www.mdpi.com/article/10.3390/microorganisms14091973/s1, Table S1. Strain information and growth of the selected colonies; Figure S1: Example of membrane filters on mPA-C agar from 10 mL wastewater effluent after 21 ± 3 h incubation and extended incubation of in total 44 ± 4 h; Figure S2: Example of membrane filters on mPA-C agar from swimming pond samples spiked with (A) wastewater influent (3 mL total volume) and (B) a mixture of six *P. aeruginosa* strains (100 mL total volume); Figure S3: Example of membrane filters on RAPID PA agar from 10 mL wastewater effluent after 21 ± 3 h incubation and extended incubation of in total 44 ± 4 h; Figure S4: Example of membrane filters on RAPID PA agar from 100 mL swimming pond samples spiked with (A) wastewater influent and (B) a mixture of six *P. aeruginosa* strains.
