# Supplementary material for: Enumeration Method for Pseudomonas aeruginosa in Water with High Bacterial Background Flora
Source: Microorganisms. 2026 Sep 7;14(9):1973. doi: 10.3390/microorganisms14091973 (PMC13609427; doi:10.3390/microorganisms14091973)
Supplement: Supplementary file 1 [file microorganisms-14-01973-s001.zip › microorganisms-4228136-supplementary.pdf]

# Supplementary Information

of

# Enumeration Method for *Pseudomonas aeruginosa* in Water with High Bacterial Background Flora

Elfy Ly [1, 2], Tess M. Hiemink [1], Sharona de Rijk [3], Karola Waar [4], Franciska M. Schets [3], Lucía Hernández Leal [1], Mark C. M. van Loosdrecht [2], Hetty Blaak [3] and Heike Schmitt [1, 2, 3]

1. Wetsus, European Centre of Excellence for Sustainable Water Technology, 8911 MA Leeuwarden, The Netherlands
2. Department Biotechnology, Delft University of Technology, 2629 HZ Delft, The Netherlands
3. Centre for Zoonoses and Environmental Microbiology, Centre for Infectious Disease Control, National Institute for Public Health and the Environment (RIVM), 3720 BA Bilthoven, The Netherlands
4. Certe Medical Microbiology, 9728 NZ Groningen, The Netherlands

Supplementary information

Table S1. Strain information and growth of the selected colonies

*Figure S1. Example of membrane filters on mPA-C agar from 10 mL wastewater effluent after 21 ± 3 hours incubation and extended incubation of in total 44 ± 4 h.*

*Figure S2. Example of membrane filters on mPA-C agar from swimming pond samples spiked with (A) wastewater influent (3 mL total volume) and (B) a mixture of six P. aeruginosa strains (100 mL total volume).*

*Figure S3. Example of membrane filters on RAPID PA agar from 10 mL wastewater effluent after 21 ± 3 hours incubation and extended incubation of in total 44 ± 4 h.*

*Figure S4. Example of membrane filters on RAPID PA agar from 100 mL swimming pond samples spiked with (A) wastewater influent and (B) a mixture of six P. aeruginosa strains.*

Table S1. Strain information and growth of the selected colonies

| Colour group | Description | mPA-C (A) | Source | Our suggestion/ analysis mPA-C (B) | Our suggestion/ analysis mPA-C (C) | Rapid (A) | Source | Our suggestion/ analysis RAPID PA (B) |
|---|---|---|---|---|---|---|---|---|
| Yellow | Yellow, light yellow, fluorescent yellow | Non target | - | Non target | Non target | Non-target | - | Non-target |
| Green | Beige/green, light green/brown, green/grey, green, dark green, light green, white/green | Target | [30,31, 36] | Target | Target | Target | [39] | Target |
| Blue | Blue, light blue, beige/blue, blue/green, light blue/green, green/blue, transparent/blue, turquoise, dark blue/green, dark green/blue | Non target | - | Non target | Non target | Target | [39] | Target |
| Brown / Tan | Light brown, brown, dark brown, brown/grey, grey/brown, blue/brown, purple/brown, green/brown, brown/green, dark brown/green, light brown/green, blue/green/brown, dark grey/brown, transparent/brown, dark blue/brown<br><br>Brown/beige, brown/yellow, yellow/brown, pink/brown, brown/pink, orange/brown, beige/brown | Target | [30, 31, 36–38] | Target | Target | Non-target | - | Target |
| Black | Black, purple/black | Target | [30, 36, 38] | Target | Target | Non-target | - | Non-target |
| Pink | Pink, light pink, pink/orange, pink/yellow, white/pink, yellow/pink, beige/pink | Non target | - | Non target | Target | Non-target | - | Non-target |
| Orange | Orange, light orange, orange/pink, orange/yellow, beige/orange, yellow/orange | Non target | - | Non target | Non target | Non-target | - | Non-target |
| Purple | Purple, purple/pink | Target | [37] | Target | Target | Non-target | - | Non-target |
| Neutral / transparent | Transparent, transparent/beige, beige, beige/transparent, white, transparent/white, transparent/yellow | Non target | - | Non target | Non target | Non-target | - | Non-target |
| Grey | Grey, grey/transparent, grey/white, light grey | Non target | - | Target | Target | Non-target | - | Non-target |

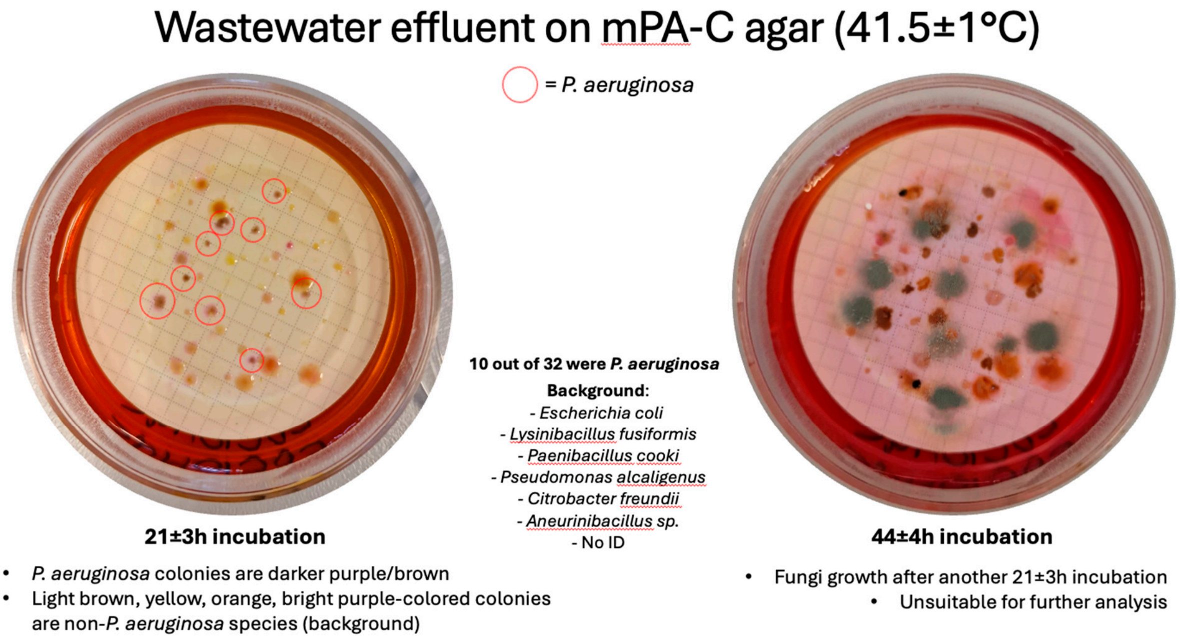


*Figure S1. Example of membrane filters on mPA-C agar from 10 mL wastewater effluent after 21 ± 3 hours incubation and extended incubation of in total 44 ± 4 h.*

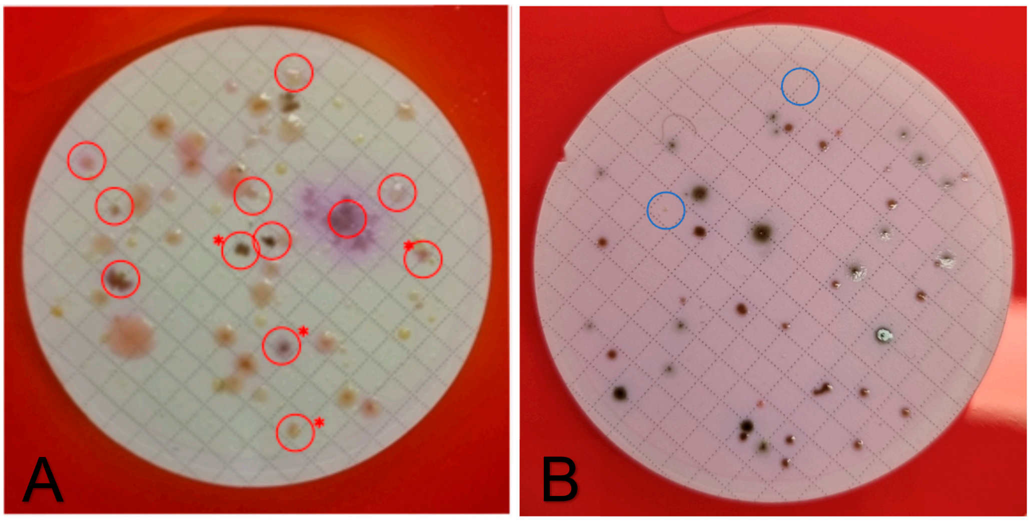


*Figure S2. Example of membrane filters on mPA-C agar from swimming pond samples spiked with (A) wastewater influent (3 mL total volume) and (B) a mixture of six P. aeruginosa strains (100 mL total volume). In panel A, red circles indicate colonies confirmed as P. aeruginosa. In panel B, all colonies were confirmed as P. aeruginosa except those marked with blue circles (the upper colony showed yellow fluorescence and was identified as an Enterococcus spp.).*

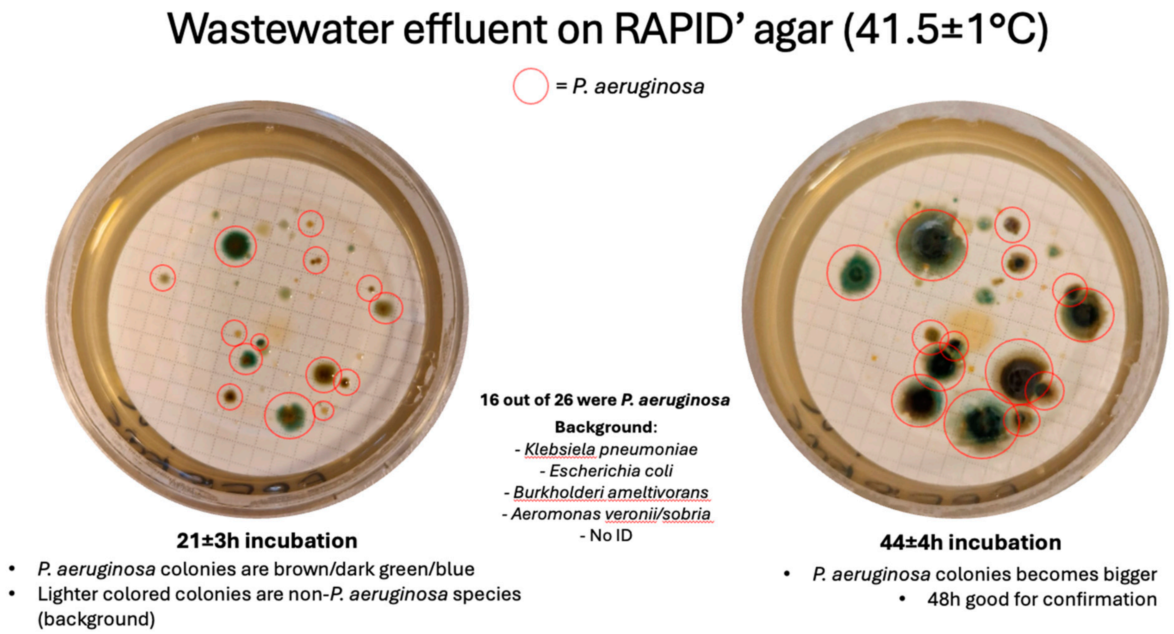


*Figure S3. Example of membrane filters on RAPID PA agar from 10 mL wastewater effluent after 21 ± 3 hours incubation and extended incubation of in total 44 ± 4 h.*

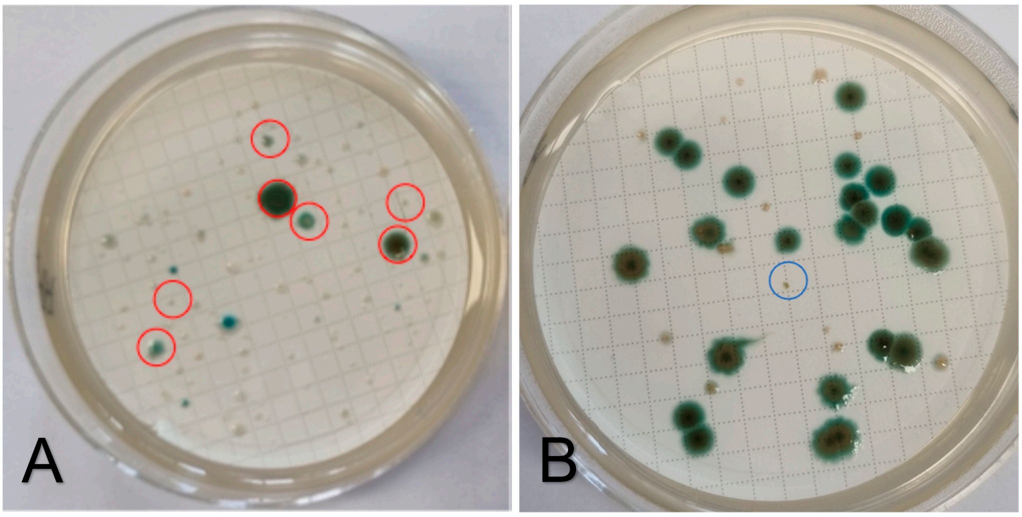


*Figure S4. Example of membrane filters on RAPID PA agar from 100 mL swimming pond samples spiked with (A) wastewater influent and (B) a mixture of six P. aeruginosa strains. In panel A, red circles indicate colonies confirmed as P. aeruginosa. In panel B, the blue circle marks a presumptive colony that did not grow upon subculturing; all remaining colonies were confirmed as P. aeruginosa (initially beige but developing dark green-blue/brown pigmentation after 44 ± 4 h, not shown in this figure).*
